# Detecting Epileptic Seizures in EEG Signals with Complementary Ensemble Empirical Mode Decomposition and Extreme Gradient Boosting

**DOI:** 10.3390/e22020140

**Published:** 2020-01-24

**Authors:** Jiang Wu, Tengfei Zhou, Taiyong Li

**Affiliations:** 1 School of Economic Information Engineering, Southwestern University of Finance and Economics, Chengdu 611130, China; wuj_t@swufe.edu.cn (J.W.); ztf10708@163.com (T.Z.); 2 Sichuan Province Key Laboratory of Financial Intelligence and Financial Engineering, Southwestern University of Finance and Economics, Chengdu 611130, China

**Keywords:** electroencephalogram (EEG), epileptic seizure detection, complementary ensemble empirical mode decomposition (CEEMD), feature selection, extreme gradient boosting (XGBoost)

## Abstract

Epilepsy is a common nervous system disease that is characterized by recurrent seizures. An electroencephalogram (EEG) records neural activity, and it is commonly used for the diagnosis of epilepsy. To achieve accurate detection of epileptic seizures, an automatic detection approach of epileptic seizures, integrating complementary ensemble empirical mode decomposition (CEEMD) and extreme gradient boosting (XGBoost), named CEEMD-XGBoost, is proposed. Firstly, the decomposition method, CEEMD, which is capable of effectively reducing the influence of mode mixing and end effects, was utilized to divide raw EEG signals into a set of intrinsic mode functions (*IMF*s) and residues. Secondly, the multi-domain features were extracted from raw signals and the decomposed components, and they were further selected according to the importance scores of the extracted features. Finally, XGBoost was applied to develop the epileptic seizure detection model. Experiments were conducted on two benchmark epilepsy EEG datasets, named the Bonn dataset and the CHB-MIT (Children’s Hospital Boston and Massachusetts Institute of Technology) dataset, to evaluate the performance of our proposed CEEMD-XGBoost. The extensive experimental results indicated that, compared with some previous EEG classification models, CEEMD-XGBoost can significantly enhance the detection performance of epileptic seizures in terms of sensitivity, specificity, and accuracy.

## 1. Introduction

Epilepsy is a common cerebral disorder. It is reported that a prevalence of 0.6%–0.8% of the global population suffers from this disease [1]. Epilepsy is generally characterized by a transient disorder of the nervous system and unpredictable occurrence [2]. Epileptic seizures generally fall into two main categories: partial and generalized [3]. The main difference between these two types of epileptic seizures lies in the occurrence region of the brain. Both epileptic seizures can occur for all races, ages, and ethnic back-grounds, but they are more common in younger and older demographics [4]. Epileptic seizures not only harm the sensory, motor, and functional aspects of the body, but they also affect the consciousness, memory, and cognition of patients [5]. Therefore, it is of great practical significance to develop an effective detection approach for epileptic seizures [6].

An electroencephalogram (EEG) is a typical measure to record the electrical activity in brains using sensors, and it can be used for epilepsy diagnosis, sleep-stage analysis, brain-computer interfaces (BCIs), and so on [7,8]. Due to it being painless and convenient, EEG is the most popular detection approach for epilepsy diagnosis [9]. The detection of epileptic seizures usually requires the manual scanning of EEG signals, which is error-prone and time-consuming [10]. Hence, it is urgent to develop effective and reliable techniques for seizure detection via EEG signals.

A variety of techniques were developed for epileptic seizure detection using EEG signals in previous research. The basic two steps involved in seizure detection using the various proposed methods are feature extraction and classification. Extracting important features from EEG signals is crucial to improving classification performance. Relevant features can be extracted from different domains, including the time domain, frequency domain, and/or time-frequency domain. There are several methods that extract time domain features for epileptic seizure detection, including amplitude and phase coupling measures [11], radial basis function neural networks based on principal component analysis (PCA) [12], fractional linear predictions [13], relative amplitude and rhythmicity [14], etc. For instance, Wei et al. extracted time domain features using amplitude and phase coupling measures based on three different coupling approaches [11]. Samanwoy et al. presented a PCA-based neural network to detect epileptic seizures [12]. Joshi et al. used fractional linear prediction to discriminate non-seizure and seizure EEG signals [13]. Murro et al. used amplitude and rhythmicity features to conduct discriminant analysis to automatically detect seizure EEGs [14].

In frequency domain analysis, the main methods include fast Fourier transform [15], higher-order spectra [16], bispectrum [17,18], power spectral analysis [19], eigenvectors [20], etc. Polat and Günes extracted the relevant features from raw EEG signals using fast Fourier transform, and built a hybrid system to detect epileptic seizures [15]. Chua et al. made a comparative study of the feature extraction from the power spectrum and the higher-order spectra, and the experimental results showed that the selected higher-order spectra features outperformed the power spectrum [16]. Ieracitano et al. extracted continuous wavelet transform (CWT) and bispectrum features, and built a number of classifiers to perform both two-way and three-way classifications [17]. Bou Assi et al. extracted quantitative features from a bispectrum, and experimental results demonstrated statistically significant differences between interictal and preictal states with bispectrum-extracted features [18]. Goldfine et al. evaluated an EEG spectrum from 4 to 24 Hz with univariate comparisons and multivariate comparisons, and the experimental results demonstrated that EEG spectral analysis could be used to demonstrate awareness in patients with severe brain injury [19]. Übeyli extracted features using eigenvector methods, and the experimental results demonstrated that the features obtained by the eigenvector methods could adequately represent EEG signals [20].

In the time-frequency domain, the main methods include wavelet transform [21], wavelet packet decomposition [22,23], multi-wavelet transform [24], Stockwell transform [25], empirical mode decomposition (EMD) [26], and so on. Bhattacharyya et al. used empirical wavelet transform to divide raw EEG signals into rhythms and extract relevant features [21]. Zhang et al. transformed EEG signals into sub-signals using wavelet packet decomposition, and wavelet packet coefficients were then fed into the autoregressive model to compute autoregressive coefficients, which were used as extracted features [23]. Peker et al. extracted relevant EEG features using a dual-tree complex wavelet transform at various levels of granularity to obtain size reduction [27]. Guo et al. used multi-wavelet transform and extracted features to identify seizures in EEG signals [24]. Kalbkhani and Shayesteh transformed raw EEG signals into the time-frequency domain using Stockwell transform, and the amplitudes of Stockwell transform in five sub-bands were extracted to construct feature vectors [25]. Pachori and Patidar employed EMD to decompose raw EEG signals, and the second-order difference plot of the decomposed components was utilized as a feature for seizure detection [26].

Apart from the time-frequency domain analysis, several approaches based on non-linear features were applied to EEG signals after decomposition. Güler et al. assessed the diagnostic performance of Lyapunov exponents in EEG signals [28]. Niknazar et al. applied recurrence quantification analysis to raw EEG signals and sub-bands for epileptic seizure detection [29]. Samanwoy et al. combined correlation dimension with standard deviation and the largest Lyapunov exponent for epileptic seizure detection [30]. Moreover, there are also some entropy-based features extracted for epileptic seizure detection, such as permutation entropy, sample entropy, approximate entropy, phase entropy, wavelet entropy, etc. These studies showed that the entropies could be utilized as effective features to develop classifiers to detect epileptic seizures.

Regarding classification, the detection results are largely decided by the performance of the selected classifiers. In previous studies, the most frequently used classifiers included decision trees [31], random forests [32], artificial neural networks [33], support vector machines [34], extreme learning machines [35], ensembles of gradient-boosted decision trees [36,37], convolutional neural networks [38], long short-term memory networks [39], etc. Among these classifiers, artificial neural networks are frequently used due to their good adaptability, generalization capability, and easy implementation [40]. Deep learning-based approaches were also applied to EEG signal classification in recent years [38,41].

From the above literature review, we can find that these previous studies commonly decompose raw EEG signals using wavelet decomposition transform, EMD, etc. and employ traditional classifiers for epileptic seizure detection. Among these popular decomposition methods, complementary ensemble empirical mode decomposition (CEEMD) succeeded in energy forecasting, fault diagnosis and others, demonstrating satisfactory performance [42,43,44,45,46]. On the other hand, as a new classification algorithm, extreme gradient boosting (XGBoost) was also applied in various fields and exhibited promising classification and prediction performance [47,48,49]. Since each of them has its own advantages, the powerful combination of CEEMD and XGBoost may potentially enhance the classification performance. Therefore, to overcome the existing weaknesses and enhance the classification performance, this study develops a detection approach of epileptic seizures using CEEMD and XGBoost, named CEEMD-XGBoost, for epileptic seizure detection. Firstly, raw EEG signals are transformed into several sub-components by CEEMD, which is capable of reducing the influence of end effects and mode mixing. Then, a set of time, frequency, time-frequency, and entropy-based features are extracted from raw signals and decomposed components, and they are further selected based on their importance scores. Finally, these extracted important features are fed into XGBoost to construct the epileptic seizure detection model. Empirically, the proposed approach was tested with the very popular Bonn dataset [50] provided by Bonn University and the recently published CHB-MIT dataset [51], provided by the Children’s Hospital Boston and Massachusetts Institute of Technology. Compared with the traditional epileptic seizure detection models, the extensive experimental results demonstrate that our proposed CEEMD-XGBoost can obtain promising detection accuracy. The main novelty of this study includes three aspects. Firstly, a novel epileptic seizure detection model that combined CEEMD with XGBoost was developed. The proposed CEEMD-XGBoost decomposes raw EEG signals into several components, then extracts various features from both raw signals and decomposed components, and finally constructs an XGBoost model to detect epileptic seizures. To our knowledge, the combination of CEEMD and XGBoost was not previously applied in epileptic seizure detection. Secondly, experiments were performed on the Bonn EEG dataset and the CHB-MIT dataset, and the extensive experiment results indicated that our proposed classification model CEEMD-XGBoost outperformed most previous models for detecting epileptic seizures. Lastly, we further evaluated some characteristics of the proposed CEEMD-XGBoost, including the impact of CEEMD and feature importance.

The main contributions of this paper are as follows: (1) we used CEEMD to decompose raw EEG signals for subsequent better feature extraction; (2) a variety of features, including time, frequency, time-frequency, and entropy-based features, were extracted from both raw signals and decomposed components; (3) XGBoost was firstly employed to construct the epileptic seizure detection model; (4) extensive experiments demonstrated the proposed CEEMD-XGBoost was very promising for epileptic seizure detection; (5) the impact of CEEMD and the importance of the selected features were further analyzed.

The rest of this paper is organized as follows: Section 2 provides a brief introduction to CEEMD and XGBoost. Section 3 describes the proposed CEEMD-XGBoost model in detail. Section 4 reports and analyzes the experimental results and discusses the proposed model. Section 5 concludes this study.

## 2. Preliminaries

### 2.1. Complete Ensemble Empirical Mode Decomposition

**Figure 1:**
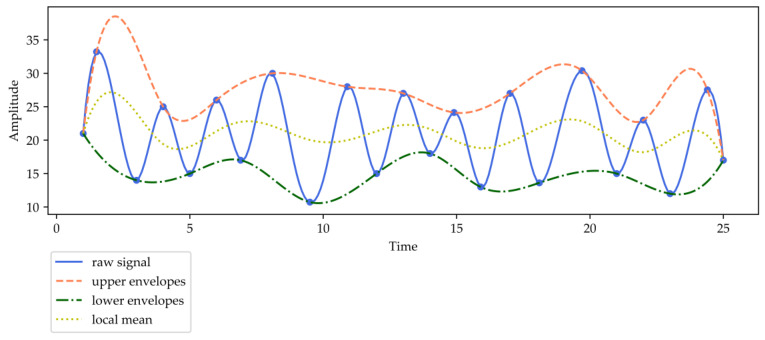
An illustration of empirical mode decomposition (EMD).

As one type of typical time-frequency analysis approach, empirical mode decomposition (EMD) [52] was proposed for time series or signal analysis fields, such as engineering, medicine, financial data analysis, etc. EMD decomposes raw time series into intrinsic mode functions (*IMF*s) and one residue. *IMF* is a function that satisfies two conditions: (1) the number of zero crossings and local extrema must either be equal to or differ at most by one; (2) the mean value of the envelope defined by the local minima and the local maxima is zero at any point. The detailed decomposition process is as follows: EMD firstly retrieves the upper and the lower envelopes which are calculated by the local extrema of the original series. Then, cubic spline is employed to construct the upper and lower envelopes by linking the local extrema. The mean of these envelopes is calculated as the first residue. Lastly, the difference between the original series and the first residue is defined as the first *IMF*. An illustration of EMD is demonstrated in Figure 1.

EMD continues to decompose the first residue into another *IMF* and one new residue. The above process repeats until the variance of the new residue is small enough to satisfy the Cauchy criterion. Finally, EMD decomposes the original series into several *IMFs* and one residue.

However, disparate scales in *IMF* components may appear in EMD mode mixing, which is defined either as a single *IMF* containing signals of wildly disparate scales or as a signal of a similar scale residing in different *IMF* components [53]. Therefore, a noise-added ensemble EMD (EEMD) was designed to cope with the problem of mode mixing [46,53,54]. Although EEMD can effectively deal with the influence of mode mixing, another problem can be caused, such as residue noise. Complete ensemble empirical mode decomposition (CEEMD) [55,56] was developed from the previous EMD and EEMD, where different noises are appended in different stages and then each mode is generated by a unique residue. CEEMD decomposes the original series with *N* different noise realizations by utilizing pairs of positive and negative white noises to generate complementary *IMFs*. CEEMD both solves the mode mixing problem and provides an exact reconstruction of the original series. Compared with wavelet decomposition, CEEMD has no resolution or harmonic complication problem [57].

By using CEEMD, a raw EEG signal can be seen as the sum of several *IMFs* and one residue *R*. Normally, The *IMF*s and residue are relatively simpler than the raw complex EEG signal. Then, we can extract a set of multi-domain features from the decomposed components. Thus, we expect that more detailed features of the decomposed components are able to contribute to enhancing the performance of epileptic seizure detection.

### 2.2. Extreme Gradient Boosting

As a kind of gradient boosting machine (GBM), XGBoost [58,59,60] is commonly employed for supervised learning problems. XGBoost follows the previous ideas in gradient boosting and constructs a “strong” learner by integrating the predictions of a group of “weak” sub-learners whose prediction performances are just a little better than random guessing. In XGBoost model, the “weak” sub-learners are generally regression trees. The combination of these “weak” sub-learners employs a gradient learning strategy. Basically, a first “weak” sub-learner is trained and, subsequently, a second sub-learner is constructed to fit the residuals of the first one. All steps of training a model to fit the residuals of the previous one are repeated until the stopping criterion is satisfied. Thus, the XGBoost model is a weighted ensemble of these individual predictions of the “weak” sub-learners. Let *I* be a molecule with a vector of *x_i_*; then, the XGBoost model can be seen as the ensemble of *K* additive functions.

(1) $${\hat{y}}_{i}=\emptyset \left(x_{i}\right)=\overset{K}{\underset{k=1}{\sum }}f_{k}\left(x_{i}\right),\;f_{k}\in ℱ,\;$$

where ℱ is a group of regression trees. The function $f_{k}$ makes the *k* prediction based on a certain output. The whole training process is to construct regression trees, including the structures of the trees and the leaf scores. In order to avoid trapping into overfitting, XGBoost should simplify the complexity of the model by decreasing the computation. Therefore, the XGBoost model is built on the loss + penalty objective function.

(2) $$Obj^{\left(t\right)}=\overset{n}{\underset{i=1}{\sum }}l\left(y_{i},{\hat{y}}_{i}\right)+\overset{k}{\underset{i=1}{\sum }}\Omega \left(f_{i}\right),$$

where *l* is a loss function that is used to measure the difference between the target $y_{i}$ and the prediction ${\hat{y}}_{i}$. Ω is used to penalize the complexity of the model, and it is calculated based on the scores of each leaf and the number of leaves. The main point of the calibration process of XGBoost model is ultimately described as follows:

(3) $$Obj^{\left(t\right)}=-\frac{1}{2}\overset{T}{\underset{j=1}{\sum }}\frac{G_{j}^{2}}{H_{j}+\lambda }+\tau ,$$

where *H* and *G* are determined by the Taylor series expansion of the loss function, *T* represents the number of leaves, and λ is the *L*2 regularization parameter.

Since XGBoost exhibits good prediction and classification accuracy [47,48,49], it was adopted to construct the epileptic seizure detection model in this study.

## 3. Methodology

### 3.1. Framework

**Figure 2:**
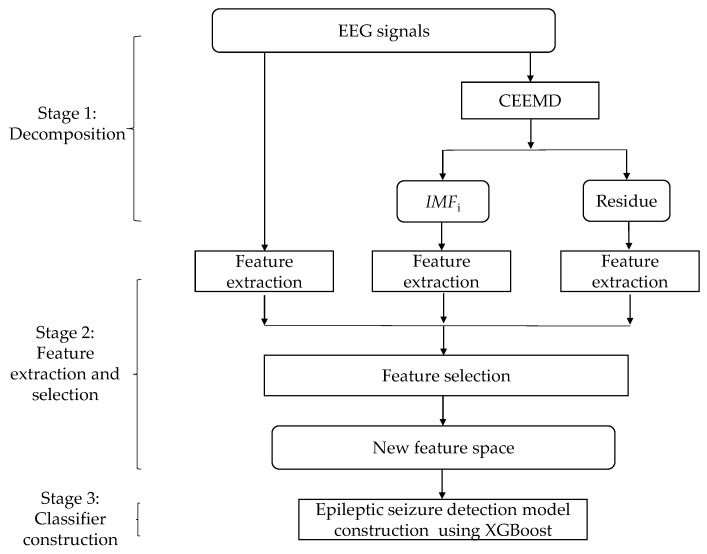
The complete ensemble empirical mode decomposition (CEEMD)/Extreme gradient boosting (XGBoost) model.

Based on multi-domain features, this study proposes an epileptic seizure classification technique that combines CEEMD with XGBoost, named CEEMD-XGBoost, to automatically detect epileptic seizures. The proposed model consists of three stages, as shown in Figure 2.

Stage 1: Decomposition. In view of the highly complex characteristics of raw EEG signals, it is hard to achieve satisfactory detection performance using raw signals. In order to better extract features from raw EEG signals, one common method is to transform raw EEG signals into sub-signals. Therefore, CEEMD is employed to divide each raw EEG signal x(n) into (1) *M IMF* components *IMF_j_* (*j* = 1, 2, …, *M*) and (2) one residue component *R*.

Stage 2: Feature extraction and selection. Since the extracted features are fed into the subsequent classification model, feature extraction is a very important step. In order to comprehensively represent the characteristics of EEG signals, multi-domain features, including time domain, frequency domain, time-frequency domain, and entropy-based features, are extracted. In addition, a large number of features may reduce the classification performance; thus, relevant features are further selected based on their importance scores.

Stage 3: Classifier construction. The above-selected features are fed into an XGBoost classification algorithm to develop the epileptic seizure detection model.

The proposed CEEMD-XGBoost employs the strategy of “divide and conquer”, which is very popular in energy forecasting, fault diagnosis, image processing, and so on [42,43,44,45,46,61,62,63]. It firstly applies CEEMD to decompose each raw signal into a set of components (several *IMF*s and one residue). Generally, the high-frequency characteristics are retained in the first *IMF*s, while the remaining *IMF*s and the residue imply the low-frequency characteristics of the raw EEG signal. Secondly, a group of multi-domain features are subsequently extracted, and then the relevant features are selected based on their importance scores. In previous research, the features were extracted from either raw EEG signals or decomposed signals. Since both raw EEG signals and decomposed signals may contain potentially useful characteristics for the subsequent classifier construction, we expect that extracting features from both raw EEG signals and decomposed signals contributes to the performance improvement of the epileptic seizure detection model. Thus, we extracted various features, including the time-domain, frequency-domain, time-frequency, and entropy-based features, from raw EEG signals and decomposed *IMF*s and residue. Feature selection is conducted using XGBoost. Finally, the relevant features are fed into an XGBoost model to construct the epileptic seizure detection model.

Significantly, some recent studies integrated decomposition and classification models to detect seizures using EEG signals. However, these previous studies differed from this study with respect to decomposition, feature extraction, and/or classification approach in that (1) they decomposed raw EEG signals using wavelet decomposition transform, EMD, etc., (2) they extracted features from either raw EEG signals or decomposed signals, and (3) they detected epileptic seizures using traditional classifiers. Previous studies demonstrated that CEEMD is superior to EMD and XGBoost shows better classification performance than traditional classifiers. In contrast, the current study uses CEEMD to divide raw EEG signals into several subseries, and further develops an epileptic seizure detection model using XGBoost based on the relevant features extracted from both raw EEG signals and decomposed subseries.

### 3.2. Dataset

To evaluate the performance of our proposed CEEMD-XGBoost, this study used two benchmark EEG datasets, including the Bonn dataset and the CHB-MIT dataset. The Bonn EEG segments were collected from the epilepsy dataset of Bonn University [50]. The EEG dataset contains five subsets (A, B, C, D, and E), and each one consists of 100 single-channel segments. These segments were selected and cut out from continuous multi-channel EEG recordings. The EEG segments of sets A and B were acquired from five healthy volunteers, who were awake with eyes open and closed. Sets C, D, and E originated from the EEG archive of presurgical diagnosis. Sets C and D were from five patients and contain only activity measured during seizure-free intervals. Set E only contains EEG segments collected from the epileptogenic zone during epileptic seizure activity. The sampling rate of Bonn EEG data is 173.61 Hz, and an EEG segment lasts for 23.6 s. Thus, each signal segment contains 173.61 × 23.6 = 4097 sampling points. More detailed information on the Bonn EEG dataset can be accessed in Reference [50]. The above five EEG subsets were utilized in the current study. Table 1 lists the summary of the Bonn EEG dataset.

**Figure 3:**
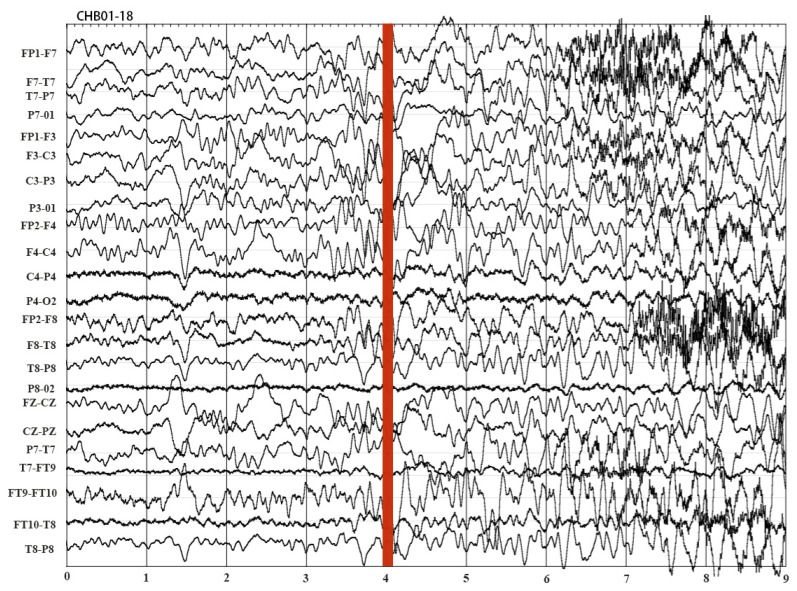
A raw multi-channel EEG signal from patient 01 in the CHB-MIT (Children’s Hospital Boston and Massachusetts Institute of Technology) dataset.

The second dataset named the CHB-MIT dataset was collected from the Children’s Hospital Boston, and it is available at PhysioNet [51]. The CHB-MIT dataset recorded the multi-channel EEG signals of 23 patients during epileptic seizure and non-seizure activity. These 23 patients included 18 females and five males from age 2–22. There are 23 channels in most EEG files and 24 channels in a few cases, and the sampling rate is 256 Hz. The segments of each seizure in EEG signals were annotated by experts. Figure 3 illustrates one segment of multi-channel EEG signals of patient 01 in the CHB-MIT dataset. As shown in Figure 3, an epileptic seizure began at the fourth second, and then the EEG signal dramatically fluctuated after the red bar.

### 3.3. EEG Signal Decomposition

**Figure 4:**
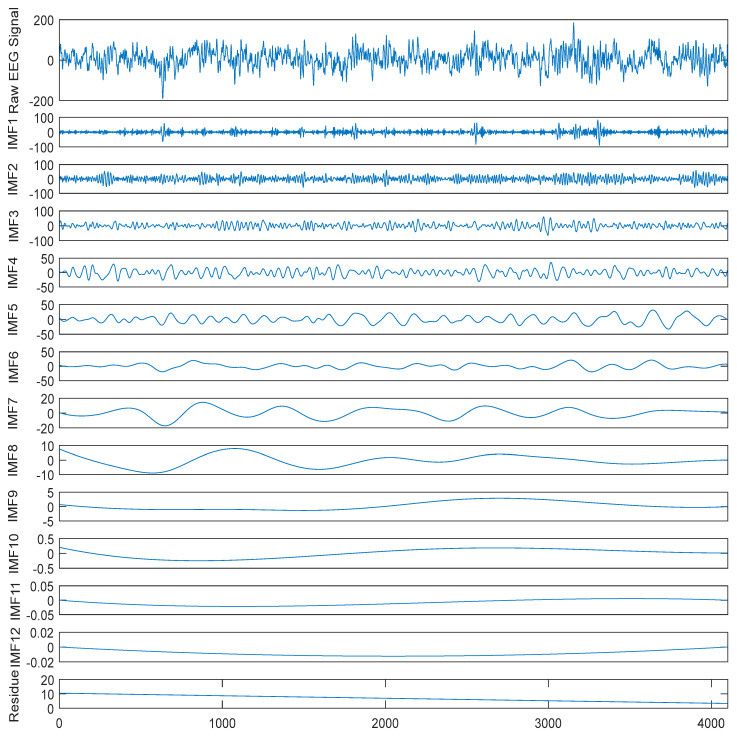
A raw EEG segment from set A in the Bonn dataset and the corresponding components decomposed by complete ensemble empirical mode decomposition (CEEMD).

**Figure 5:**
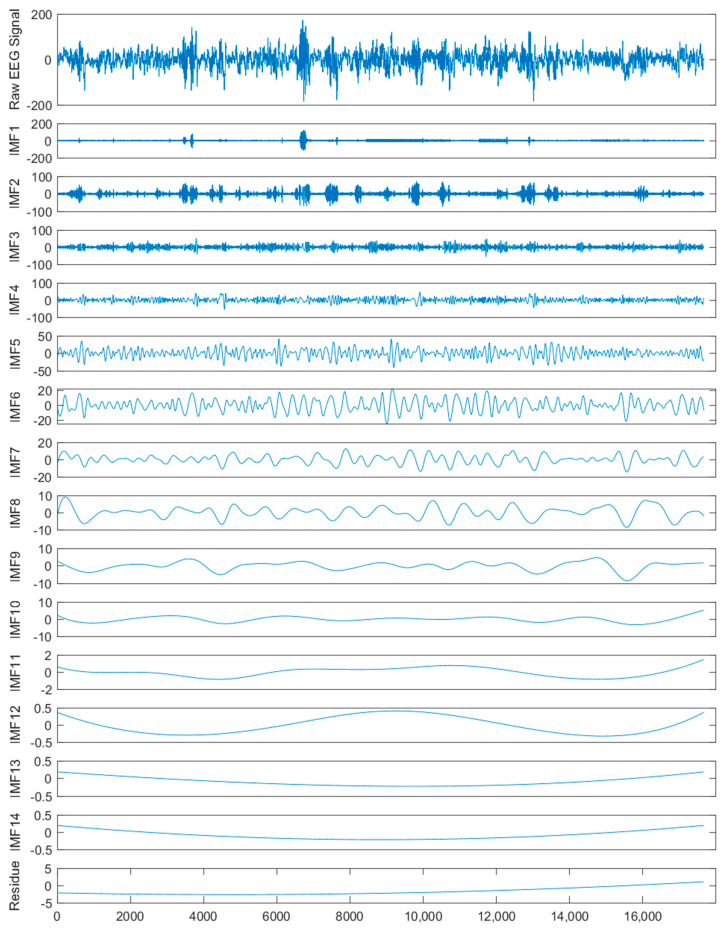
A raw EEG segment from patient 01 in the CHB-MIT dataset and the corresponding components decomposed by complete ensemble empirical mode decomposition (CEEMD).

Because of the complexity of raw signals, decomposition methods are commonly used to decompose raw signals for better performance of prediction and classification in the field of signal processing [22]. This idea can also be utilized in epileptic seizure detection because of the nonlinearity and nonstationary of EEG signals. We employed CEEMD to decompose each raw EEG signal into several *IMF*s and one residue according to amplitude and frequency in this study. Then, these raw EEG signals and the decomposed components were considered for the subsequent feature extraction. As an example, the two raw EEG segments and corresponding components decomposed using CEEMD from set A in the Bonn dataset and patient 01 in the CHB-MIT dataset are illustrated in Figure 4 and Figure 5, respectively.

It can be seen from these two figures that the decomposed subseries are simpler than the raw EEG signals, which is probably helpful for the feature extraction and the subsequent classification.

### 3.4. Feature Extraction

Feature extraction is important for representing nonstationary and nonlinear EEG signals. To potentially enhance the detection performance, we extract multi-domain features from raw EEG signals, the decomposed *IMF*s, and residues, which can more comprehensively represent the characteristics of EEG signals. The aforementioned studies used either time-frequency domain or entropy domain features for epileptic seizure detection. In this work, we employed three Python packages called Tsfresh [64], Entropy (https://github.com/raphaelvallat/entropy), and pyEntropy (https://github.com/nikdon/pyEntropy) to extract four categories of features from both raw EEG signals and the decomposed components: (1) time domain features; (2) frequency domain features; (3) time-frequency domain features; (4) entropy-based features. Specifically, Tsfresh conducted the feature extraction process using 63 time series characterization methods, which calculated a total of 794 descriptive time series features, including the above four categories of features. Additionally, we extracted four entropy-based features using Entropy and pyEntropy Python packages. Thus, we extracted 798 features in total for a time series. Since a raw Bonn EEG signal was decomposed into 12 *IMF*s and one residue, and a raw CHB-MIT EEG signal into 14 *IMF*s and one residue, we totally extracted 798 × 14 = 11,172 features for the Bonn dataset and 798 × 16 = 12,768 features for the CHB-MIT dataset.

#### 3.4.1. Time Domain, Frequency Domain, and Time-Frequency Domain Features

Previous research showed that extracting features from different domains, including time domain, frequency domain, and/or time-frequency domain, is effective for developing epileptic seizure detection models [11,12,13,14,15,16,17,18,19,20,21,23,24,25,26]. Although these three types of features were proposed, none are able to comprehensively characterize EEG signals. Therefore, the combination of all of these features has the potential to improve the classification performance. In this study, time domain, frequency domain, and time-frequency domain features were extracted using Tsfresh Python packages, which are listed in Table 2. The complete list of the 794 descriptive time series features is available in Reference [64].

#### 3.4.2. Entropy-Based Features

Entropy is commonly used to measure the amount of disorder in the system [65]. It can be utilized to measure the randomness of signals and to analyze complex EEG signals. We totally extracted six entropy-based features including permutation entropy, Shannon entropy, spectral entropy, approximate entropy, sample entropy, and singular value decomposition entropy.

• Permutation entropy

Permutation entropy (PE), which was introduced by Christoph and Bernd [66], is utilized to measure the complexity of time series through comparing neighboring values. It can be calculated as follows [67]:

(4) $$\mathrm{PE}=-\overset{n}{\underset{k=1}{\sum }}s_{k}\log s_{k},$$

(5) $$s_{k}=\frac{t_{k}}{N-m+1},$$

where *N* represents the length of the decomposed signal, $t_{k}$ is the occurrence of *k*-th symbol, $s_{k}$ indicates the probability of occurrence of the *k*-th permutation in the time series, and *n* implies permutation order of n≥2. In this study, we chose the embedding dimension *m* of 3 and delay of 1.

• Shannon entropy

Shannon entropy (ShE) is a standard measure of sequential state, and it can be used to estimate the average minimum number of bits required for symbol coding in terms of the frequency of the symbol [32]. It can be expressed as

(6) $$\mathrm{ShE}=-\overset{N}{\underset{i=1}{\sum }}p\left(i\right)\log_{2}p\left(i\right),$$

where *i* represents all observed values of EEG series data, and *p*(*i*) represents the probability that value occurs in the whole EEG series.

• Spectral entropy

Because of the difference of signal intensity among individuals, the absolute values may vary from individual to individual, but Shannon entropy is not standardized as the total power of EEG signals [68]. In order to overcome this shortcoming, spectral entropy (SpE) is adopted in this study. Spectral entropy is defined to be the Shannon entropy of the power spectral density (PSD) of the data. It can be expressed as follows:

(7) $$\;\mathrm{SpE}=-\underset{f=0}{\sum }p_{f}\log p_{f},$$

where *p_f_* is the relative power of the component with frequency *f*. *f* was set to 100 in this study.

• Approximate entropy

Approximate entropy (ApE) is utilized to quantify the unpredictability of fluctuations and the regularity of time series. A smaller value means that the data perform well in terms of regularity and prediction [69]. It can be expressed as follows:

(8) $$\mathrm{ApE}=\varphi^{m}\left(r\right)-\varphi^{m+1}\left(r\right),$$

(9) $$\varphi^{m}\left(r\right)=\frac{1}{N-\left(m-1\right)\tau }\overset{N-\left(m+1\right)\tau }{\underset{i=1}{\sum }}logC_{i}^{m}\left(r\right),$$

(10) $$C_{i}^{m}\left(r\right)=\frac{1}{N-\left(m-1\right)\tau }\overset{N-\left(m+1\right)\tau }{\underset{i=1}{\sum }}\theta \left(r-d\left(x\left(i\right),\;x\left(j\right)\right)\right),$$

(11) $$d\left(x\left(i\right),x\left(j\right)\right)=\underset{k=1,2,\ldots ,m}{\max}\left|y\left(i+\left(k-1\right)\tau \right)-y\left(j+\left(k-1\right)\tau \right)\right|,$$

where *m*, *r*, *τ*, and *N* represent the embedding dimension, similarity coefficient, time delay, and number of data points, respectively. The correlation dimension is computed by Equation (10). When *x* is smaller than 0, the value of *θ*(*x*) is equal to 0. d(x(i), x(j)) measures the distance by Equation (11). In this work, we chose *m* = 2, *r* = 0.15 times the standard deviation of the EEG signal, and *τ =* 1.

• Sample entropy

Sample entropy (SaE) is derived from approximate entropy and is used to assess the complexity of physiological time series signals [70]. In the aspect of trouble-free implementation and data length independence, it does better than ApE. SaE can be expressed as

(12) $$\mathrm{SaE}=-\ln\frac{A^{m}\left(r\right)}{B^{m}\left(r\right)},$$

where $B^{m}\left(r\right)$ represents the probability of matching two sequences for *m* points, while $A^{m}\left(r\right)$ indicates the probability of matching two sequences for *m* + 1 points. *m*, *r*, and *τ* were set to 2, 0.2 times the standard deviation of the EEG signal, and 1 in this study, respectively.

• Singular value decomposition entropy

Singular value decomposition entropy (SvdE) is an indicator of the number of eigenvectors needed to fully interpret the data set. In other words, it measures the dimensions of data. It can be calculated as follows:

(13) $$\mathrm{SvdE}=-\overset{M}{\underset{i=1}{\sum }}\bar{\sigma_{i}}log_{2}\left(\bar{\sigma_{i}}\right),$$

(14) $$Y={\left[y_{1},y_{2},\ldots ,y_{\left(N-\left(r-1\right)\tau \right)}\right]}^{T},$$

(15) $$y_{i}=\left[x_{i},\;x_{i+\tau },\ldots ,x_{i+\left(r-1\right)\tau }\right],$$

where *M* represents the number of singular values of the embedded matrix Y, which can be obtained by Equation (14). $\sigma_{1}$, $\sigma_{2}$, …, $\sigma_{M}$ are the normalized singular values of Y. *r* indicates the order of permutation entropy, and *τ* represents the time delay, which were respectively set to 3 and 1 in this study.

### 3.5. Classification and Performance Evaluation

Since each EEG signal was decomposed into several *IMF*s and one residue, we extracted a large number of features from raw EEG signals and the decomposed ones. Due to the large number of features, performing classification in such a high-dimensional feature space may influence the classification performance. In addition, the feature space may contain some irrelevant features, which reduces the classification performance and increases the computing cost. Feature selection plays a very key role in classifier training, which chooses the best subset of features from all the extracted features. In this study, redundant features were removed due to their low importance scores in XGBoost, and the threshold of the importance scores was set to 0.001. Since XGBoost shows better classification performance than traditional classifiers, it was chosen as the classifier for epileptic seizure detection. Thus, the pruned features were fed into the subsequent classifier XGBoost.

To accurately evaluate the classification performance and decrease the potential bias of training and testing data, *k*-fold cross-validation was employed in this study. Generally, the detection performance is evaluated by three main statistical measurements of sensitivity (SEN), specificity (SPE), and accuracy (ACC).

(16) $$SEN=\frac{TP}{TP+FN}\times 100.00\%,$$

(17) $$SPE=\frac{TN}{TN+FP}\times 100.00\%,$$

(18) $$ACC=\frac{TP+TN}{TP+TN+FP+FN}\times 100.00\%,$$

where *TP* is true positive, *FP* is false positive, *TN* is true negative, and *FN* is false negative.

## 4. Experimental Results

### 4.1. Experimental Settings

**Figure 6:**
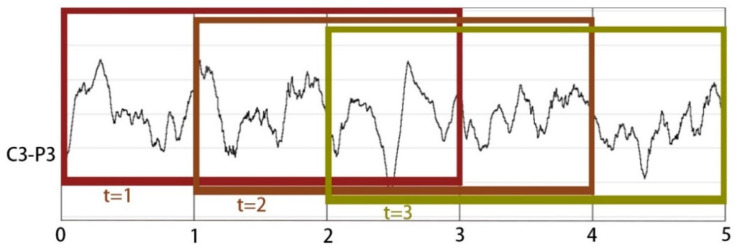
An example of the CHB-MIT EEG segmentation.

To compare the performance of our proposed methodology with previous research, two benchmark EEG datasets, including the Bonn dataset and the CHB-MIT dataset, were used in this study. Since the Bonn EEG dataset consists of five subsets, the various cases of the Bonn dataset were considered as shown in Table 3. In addition, to further assess the performance of CEEMD-XGBoost in discriminating non-seizure and seizure, we applied the proposed method to a larger dataset named the CHB-MIT EEG dataset. Because the original CHB-MIT signals are not directly segmented into sub-series of non-seizure or seizure states, we manually divided them into a collection of overlapped fragments with a fixed length. Following previous research [71,72], with a three-second sliding window, we split both seizure and non-seizure segments from five patients who were randomly selected in the CHB-MIT dataset, and we eventually obtained an EEG segment dataset, including 2675 epileptic seizure segments and 2675 non-seizure segments. Since each EEG signal contains 23 channels and the sampling rate is 256 Hz, the three-second EEG fragment included 17,664 sampling points. In general, the CHB-MIT EEG segment dataset has 5350 23-channel EEG segments, including 2675 seizure segments and 2675 seizure-free ones, and each segment consists of 17,664 sampling points. Figure 6 illustrates the process of the CHB-MIT EEG signal segmentation. The Bonn dataset and the CHB-MIT dataset used in this article are publicly accessible EEG datasets. This article does not contain any studies with human participants performed by any of the authors.

Thus, our subsequent epileptic seizure detection was conducted on these EEG fragments, as shown in Table 3.

In order to better estimate the performance of our proposed method, *k*-fold cross-validation (*k* = 10) was employed. The entire dataset was evenly divided into 10 subsets. Each subset was used for testing the model once and for training the model nine times. The performance was estimated by averaging the performances derived in all the 10 cases of cross-validation.

All experiments were performed using Python 3 on a 64-bit Microsoft Windows 10 with an i7-8565U 1.8 GHz central processing unit (CPU) and 8 GB of random-access memory (RAM).

### 4.2. Results and Analysis

#### 4.2.1. Experimental Results of the Proposed Methodology

In this study, each EEG segment was firstly transformed into several *IMF*s and one residue using CEEMD. Specifically, in consideration of the length of raw EEG signal, each EEG segment in the Bonn dataset was decomposed into 12 *IMF*s and one residue, and each EEG segment in the CHB-MIT dataset was divided into 14 *IMF*s and one residue. Then, the relevant features were extracted from multi-domains, and the optimal feature subset was further selected. The XGBoost classifier was developed to detect epileptic seizures in EEG signals. Table 4 reports the classification results of 10-fold cross-validation of the 13 cases in the Bonn dataset and the CHB-MIT dataset, respectively.

For each case in Table 4, the corresponding classification performance declined with the increasing number of categories. As for the Bonn dataset, it can be observed that two-class classification cases showed the best performance among all cases

For the CHB-MIT dataset, our proposed method performed worse in terms of classification accuracy than the Bonn dataset. Compared with the single channel signals, the multi-channel ones contain more information and are more complex. Although the multi-channel EEG records contain more information of epileptic seizure, there may be some channels which are irrelevant and redundant. Therefore, it is possible to lead to a decrease in detection performance.

As for the execution time of 10-fold cross-validation, it increased with the increase in size of the dataset. The execution time was between 10.2 s and 85.1 s on the Bonn dataset and 772.0 s on the CHB-MIT dataset, showing that the epileptic seizure detection model can be built in a relatively short time period.

In general, the proposed CEEMD-XGBoost achieved promising detection performance, and the detection accuracies were higher than or equal to 99.00% in all 12 cases in the Bonn dataset and 95.79% in the CHB-MIT dataset.

#### 4.2.2. Classification Performance Comparison with State-of-the-Art Seizure Detection Methods

To better assess the classification performance of our proposed methodology, it was compared with some of the previous methods using the Bonn EEG dataset and the CHB-MIT EEG dataset. The classification accuracies of the proposed approach and the existing techniques for various classification cases are listed in Table 5, Table 6, Table 7 and Table 8. The experimental results demonstrated that the proposed method achieved the highest detection accuracies in most cases compared with the previous methods.

As observed in cases {A, E}, {B, E}, {D, E}, and {A, D}, the proposed method in this study achieved the perfect ACC of 100.00%. In case {C, E}, our proposed method obtained the ACC of 99.50%, which was higher than most of the exhibited methods. For the non-ictal-ictal cases, the proposed method in this study reached the highest ACC in cases {AB, E}, {ACD, E}, and {BCD, E}. In cases {CD, E} and {ABCD, E}, the proposed method achieved a higher ACC than most of the exhibited methods. For instance, the proposed method achieved the fourth highest ACC among all 12 methods in case {CD, E} and the fourth highest ACC among all 15 methods in case {ABCD, E}. For the non-seizure-interictal-ictal cases, the proposed method achieved the highest ACC in case {A, D, E}. In case {AB, CD, E}, the proposed method achieved the third highest ACC among all seven methods.

Table 8 shows that our proposed method achieved the highest classification accuracy on the CHB-MIT dataset. On one hand, based on the same decomposition method (i.e., CEEMD), XGBoost obtained the highest classification accuracy compared with neural network (NN), support vector machine (SVM), and random forest (RF). On the other hand, our proposed method also obtained the highest classification accuracy compared with some previous studies.

In summary, CEEMD-XGBoost outperformed most exhibited methods. The proposed CEEMD-XGBoost obtained the highest classification accuracy in eight classification cases (i.e., cases {A, E}, {B, E}, {D, E}, {A, D}, {AB, E}, {ACD, E}, {BCD, E}, and {A, D, E}) and relatively high classification accuracy in the remaining four classification cases (i.e., {C, E}, {CD, E}, {ABCD, E}, and {AB, CD, E}) on the Bonn dataset, while it achieved the highest classification accuracy on the CHB-MIT dataset. Therefore, CEEMD-XGBoost is a promising approach for epileptic seizure detection.

### 4.3. Discussion

To more comprehensively investigate our proposed CEEMD-XGBoost, we further discuss some characteristics of the proposed model for epileptic seizure detection, including the impact of CEEMD and the importance of features.

#### 4.3.1. The Impact of CEEMD

On the basis of the same feature extraction, feature selection, and classification method (XGBoost), we evaluated the impact of CEEMD on the detection performance. Table 9 reports the corresponding classification results of 10-fold cross-validation with and without CEEMD on the Bonn dataset and the CHB-MIT dataset.

Table 9 demonstrates that the proposed method CEEMD-XGBoost outperformed the XGBoost without CEEMD in all cases and achieved better classification performance. The results show that CEEMD had a significant positive impact on the classification performance, indicating that extracting features from the decomposed signal can improve the detection performance.

#### 4.3.2. The Importance of the Selected Features

To gain deep insight into the importance rank of individual features for epileptic seizure detection, which was rarely examined in previous literature, we selected XGBoost for feature ranking and pruning. An advantage of using XGBoost is that it is relatively straightforward to calculate importance scores for each feature after the boosted trees are built. Generally, feature importance provides a score that represents how important each feature is in the boosted decision trees. The more a feature is employed to make key decisions using decision trees, the higher its relative importance score becomes. For a single decision tree, the importance is calculated by the amount that each feature split point improves the performance measure. The feature importance is eventually averaged across all of the decision trees in the XGBoost model. Since the importance is computed for each feature, features can be ranked and compared to each other.

To comprehensively assess the importance of features on the two datasets, we performed a feature ranking based on five classifications (A-B-C-D-E) on the Bonn dataset and two classifications (Non-seizure-seizure) on the CHB-MIT dataset using XGBoost. According to the importance scores of features, we rank the input features and list the 20 most important features along with their relative importance scores in Table 10 and Table 11.

From Table 10, we can see that the 20 most important features on the Bonn dataset came from different feature categories. Among the top 20 features, 13, three, three, and one features belonged to the categories of time-domain, frequency, entropy-based, and time-frequency, respectively, indicating that the multi-domain features contributed to epileptic seizure detection. On the other hand, we find that the top 20 features were extracted from different components, including the raw signal, *IMF*_1_, *IMF*_2_, *IMF*_3_, *IMF*_4_, and *IMF*_10_, which further validated the effect of EEG signal decomposition. In other words, decomposing each raw EEG signal into several components (*IMF*s and one residue) contributed to extracting more comprehensive features.

From Table 11, we can see something similar to Table 10. Among the top 20 features, 14, four, one, and one features belonged to the categories of time-domain, frequency, entropy-based, and time-frequency, respectively, and these features came from the raw EEG signal, *IMF*_1_, *IMF*_2_, *IMF*_3_, *IMF*_4_, *IMF*_5_, *IMF*_7_, and *IMF*_8_, which further confirmed the effectiveness of feature extraction and signal decomposition.

In summary, decomposing raw EEG signals into sub-components benefits the extracted features in representing raw EEG signals. The better classification performance can be attributed to the highly discriminative features. The extracted multi-domain features better represent nonstationary and nonlinear EEG signals. Furthermore, the XGBoost classifier has a superior classification capability to other classifiers. Hence, the above three main factors led to the satisfactory classification accuracy in our work.

## 5. Conclusions

It is a big challenge to accurately detect epileptic seizures due to the complexity of EEG signals. For the purpose of better detecting epileptic seizures using EEG signals, this paper proposed a novel epileptic seizure detection approach integrating CEEMD and XGBoost. Firstly, CEEMD was utilized to decompose each raw EEG signal into a collection of *IMF*s and one residue. Then, a group of multi-domain features were extracted from both raw signals and decomposed components, and they were further chosen according to the importance of the extracted features. Finally, XGBoost was applied to develop a classification model to detect seizure-free and seizure EEG signals. To the best of our knowledge, this is the first application of a combination of CEEMD and XGBoost to epileptic seizure detection. The extensive experimental results demonstrate that (1) compared with some state-of-the-art classification models, the CEEMD-XGBoost model can significantly enhance the detection performance of epileptic seizure in EEG signals, (2) by decomposing raw EEG signals into sub-components, we can better extract features to represent raw EEG signals, and (3) individual multi-domain features have different levels of importance for the classification performance, and the most important features come from multiple domains.

Since a large number of features are extracted and input into the classifier for epileptic seizure detection, the proposed CEEMD-XGBoost may need higher computing cost than single-domain feature methods. Future work could be extended in three aspects: (1) extracting more effective features to build epileptic seizure detection models, such as bispectrum features, etc.; (2) further investigating and comparing the contribution of different categories of features; (3) evaluating the scaling ability of CEEMD-XGBoost using more EEG data, such as the TUH (Temple University Hospital) EEG epilepsy corpus, etc.

## Figures and Tables

**Table 1:** Summary of the Bonn electroencephalogram (EEG) data.

	Set A	Set B	Set C	Set D	Set E
Volunteer type	Heathy	Heathy	Epileptic	Epileptic	Epileptic
Volunteer state	Awake state with eyes open	Awake state with eyes closed	Interictal	Interictal	Ictal
Number of channels	100	100	100	100	100
Electrode placement	International 10–20 system	International 10–20 system	Hippocampus opposite to hemisphere	Within epileptogenic zone	Within epileptogenic zone

**Table 2:** A subset of the extracted features.

Category	Sub-Category	Features
Time domain	Energy	Absolute energy, energy ratio
	Autocorrelation	Mean and variance over the autocorrelation for different lags Partial autocorrelation
	Autoregression	Autoregressive coefficients
	Linear trend intercept	Correlation coefficient, *p*-value, intercept, slope, standard error
	Statistics	Mean absolute change Spectral density estimationSignal symmetry Maximum, minimum, mean, median, sum, standard deviation, variance, quantile, duplication number of crossings, number of peaks, etc.
Frequency domain	Fourier transform spectrum	Fourier transform aggregate and coefficients
Time-frequency domain	Wavelet	Continuous wavelet coefficients and peaks

**Table 3:** Various cases considered in this study.

Dataset	Cases	Classes	Description	Type
Bonn	I	A-E	Non-seizure (eyes open) and ictal	Two
	II	B-E	Non-seizure (eyes closed) and ictal	Two
	III	C-E	Interictal and ictal	Two
	IV	D-E	Interictal and ictal	Two
	V	A-D	Non-seizure (eyes open) and interictal	Two
	VI	AB-E	Non-seizure and ictal	Two
	VII	CD-E	Interictal and ictal	Two
	VIII	ACD-E	Non-ictal and ictal	Two
	IX	BCD-E	Non-ictal and ictal	Two
	X	ABCD-E	Non-ictal and ictal	Two
	XI	A-D-E	Non-seizure, interictal and ictal	Three
	XII	AB-CD-E	Non-seizure, interictal and ictal	Three
CHB-MIT	XIII	Non-seizure–Seizure	Non-ictal and ictal	Two

**Table 4:** Classification performance (in %) of proposed methodology using 10-fold cross-validation. SEN—sensitivity; SPE—specificity; ACC—accuracy.

Dataset	Cases	Classes	SEN	SPE	ACC	Time
Bonn	I	A-E	100.00 ± 0.00	100.00 ± 0.00	100.00 ± 0.00	10.4s
	II	B-E	100.00 ± 0.00	100.00 ± 0.00	100.00 ± 0.00	10.2s
	III	C-E	99.00 ± 0.06	100.00 ± 0.00	99.50 ± 0.03	10.3s
	IV	D-E	100.00 ± 0.00	100.00 ± 0.00	100.00 ± 0.00	10.2s
	V	A-D	100.00 ± 0.00	100.00 ± 0.00	100.00 ± 0.00	10.4s
	VI	AB-E	100.00 ± 0.00	100.00 ± 0.00	100.00 ± 0.00	14.0s
	VII	CD-E	99.00 ± 0.06	100.00 ± 0.00	99.33 ± 0.03	14.3s
	VIII	ACD-E	100.00 ± 0.00	99.67 ± 0.02	99.75 ± 0.02	18.3s
	IX	BCD-E	99.00 ± 0.06	99.33 ± 0.03	99.50 ± 0.02	19.6s
	X	ABCD-E	99.00 ± 0.06	99.50 ± 0.02	99.6 ± 0.02	23.6s
	XI	A-D-E	-	-	100.00 ± 0.00	42.4s
	XII	AB-CD-E	-	-	99.00 ± 0.04	85.1s
CHB-MIT	XIII	Non-seizure–Seizure	95.70 ± 0.06	95.89 ± 0.10	95.79 ± 0.05	772.0s

**Table 5:** Accuracy (in %) comparison with some of the existing techniques for cases I–V on the Bonn dataset.

Authors	Year	Methods	A-E	B-E	C-E	D-E	A-D
Kumar et al. [73]	2014	DWT + SVM	100.00	100.00	99.60	95.85	-
Sharmila and Geethanjali [74]	2016	DWT + KNN	100.00	98.25	97.25	95.62	-
		DWT + NB	100.00	99.25	99.62	95.12	-
Hassan and Subasi [75]	2016	CEEMDAN + LPBoost	100.00	-	100.00	97.00	-
Swami et al. [76]	2016	DTCWT + GRNN	100.00	100.00	100.00	99.50	-
Tawfik et al. [77]	2016	WPE + SVM	99.50	85.00	93.50	96.50	-
Sharma et al. [78]	2017	ATFFWT + LS-SVM	100.00	100.00	99.00	98.50	-
Jaiswal and Banka [79]	2017	LNGP + ANN	99.82	99.25	99.02	98.18	99.90
		1D-LGP + ANN	99.80	98.92	99.10	99.07	99.37
Tiwari et al. [80]	2017	Keypoint based LBP + SVM	100.00	-	-	-	-
Kaur and Singh [81]	2017	EMD + spike parameters + ANN	100.00	100.00	100.00	99.00	-
Li et al. [82]	2018	CWT + GMM + GLCM + relief + SVM	100.00	-	100.00	-	-
Singh and Dehuri [83]	2018	DWT + MLPNN	99.50	97.00	98.51	100.00	-
Zhang et al. [84]	2018	WPD + FDE + KNN	100.00	99.95	99.86	99.39	-
Gupta and Pachori [85]	2019	FBSE based rhythms + WMRPE + LS-SVM	99.50	99.50	99.50	97.50	-
Mamli and Kalbkhani [86]	2019	FSST + GLCM + SVM	100.00	99.38	99.54	96.48	-
Raghu et al. [87]	2019	Matrix determinant + MLP	99.45	96.06	97.60	97.60	-
Proposed method	2019	CEEMD + XGBoost	100.00	100.00	99.50	100.00	100.00

DWT: discrete wavelet transform; SVM: support vector machine; KNN: k-nearest neighbor; NB: naive Bayes; CEEMDAN: complete ensemble empirical mode decomposition with adaptive noise; LPBoost: linear programming boosting; DTCWT: dual-tree complex wavelet transform; GRNN: general regression neural network; WPE: weighted permutation entropy; ATFFWT: analytic time-frequency flexible wavelet transform; LS-SVM: least squares support vector machine; LNGP: local neighbor gradient pattern; ANN: artificial neural network; 1D-LGP: one-dimensional local gradient pattern; GMM: Gaussian mixture model; GLCM: gray-level co-occurrence matrix; MLPNN: multilayer perceptron neural network; WPD: wavelet packet decomposition; FDE: fuzzy distribution entropy; FBSE: Fourier–Bessel series expansion; WMRPE: weighted multiscale Renyi permutation entropy; FSST: Fourier synchro-squeezed transform; MLP: multi-layer perceptron; CEEMD: complementary ensemble empirical mode decomposition; XGBoost: extreme gradient boosting.

**Table 6:** Accuracy (in %) comparison with some of the existing techniques for cases VI–X on the Bonn dataset.

Authors	Year	Methods	AB-E	CD-E	ACD-E	BCD-E	ABCD-E
Kumar et al. [73]	2014	DWT + SVM	-	-	98.80	-	97.38
Sharmila and Geethanjali [74]	2016	DWT + KNN	98.83	96.08	96.80	96.37	97.10
		DWT + NB	99.16	98.75	97.31	95.10	95.85
Hassan and Subasi [75]	2016	CEEMDAN + LPBoost	-	-	-	-	99.20
Sharma et al. [78]	2017	ATFFWT + LS-SVM	100.00	98.67	-	-	99.20
Jaiswal and Banka [79]	2017	LNGP +ANN	-	98.88	-	-	98.72
Tiwari et al. [80]	2017	Keypoint-based LBP + SVM	-	99.45	-	-	99.31
Kaur and Singh [81]	2017	EMD + spike parameters + ANN	-	-	-	-	99.80
Singh and Dehuri [83]	2018	DWT + MLPNN	89.00	99.33	98.00	95.75	95.60
Zhang et al. [84]	2018	WPD + FDE + KNN	99.98	99.58	-	-	99.71
Zhang et al. [88]	2018	GST + SVD-based features + RF	-	99.12	-	-	99.63
Gupta and Pachori [85]	2019	FBSE-based rhythms + WMRPE + LS-SVM	-	99.00	-	-	98.60
Mamli and Kalbkhani [86]	2019	FSST + GLCM + SVM	99.73	99.59	-	-	97.38
Raghu et al. [87]	2019	Matrix determinant + MLP	97.10	96.85	96.00	-	97.20
Proposed method	2019	CEEMD + XGBoost	100.00	99.33	99.75	99.50	99.60

GST: generalized Stockwell transform; SVD: singular value decomposition; RF: random forest.

**Table 7:** Accuracy (in %) comparison with some of the existing techniques for cases XI–XII on the Bonn dataset.

Authors	Year	Methods	A-D-E	AB-CD-E
Hassan and Subasi [75]	2016	CEEMDAN + LPBoost	97.60	
Tawfik et al. [77]	2016	WPE + SVM	97.50	-
Jaiswal and Banka [79]	2017	LNGP + ANN	98.22	-
Tiwari et al. [80]	2017	Keypoint-based LBP + SVM	-	98.80
Kalbkhani and Shayesteh [25]	2017	ST + NN	99.37	99.54
Zhang et al. [84]	2018	WPD + FDE + LNN	99.39	98.76
Zhang et al. [88]	2018	GST + SVD-based features + RF	99.03	98.62
Mamli and Kalbkhani [86]	2019	FSST + GLCM + SVM	99.67	99.26
Raghu et al. [87]	2019	Matrix determinant and MLP	-	96.50
Proposed method	2019	CEEMD + XGBoost	100.00	99.00

ST: Stockwell transform.

**Table 8:** Accuracy (in %) comparison with some of the existing techniques for cases XIII on the CHB-MIT dataset.

Authors	Year	Methods	Non-Seizure-Seizure
-	-	CEEMD + NN	89.18
-	-	CEEMD + SVM	90.07
-	-	CEEMD + RF	90.90
Rafiuddin et al. [89]	2011	WT + LDA	80.16
Khan et al. [90]	2012	DWT + LDA	91.80
Behnam et al. [91]	2015	DWT + SLMM + MLP + KNN	90.00
Zabihi et al. [92]	2016	PSR + LDA + NB	94.69
Yuan et al. [72]	2018	WT + CtxFusion EEG	95.71
Wei et al. [93]	2019	CNN + MIDS	84.00
Proposed method	2019	CEEMD + XGBoost	95.79

NN: neural network; RF: random forest; WT: wavelet transform; LDA: linear discriminant analysis; SLMM: singular Lorenz measures method; PSR: phase-space reconstruction; NB: naive Bayes; CtxFusion EEG: wavelet transform context fusion EEG; CNN: convolutional neural network; MIDS: merger of increasing and decreasing sequences.

**Table 9:** Performances (in %) of detection models with CEEMD vs. without CEEMD.

Dataset	Cases	Classes	XGBoost			CEEMD-XGBoost		
			SEN	SPE	ACC	SEN	SPE	ACC
Bonn	I	A-E	100.00 ± 0.00	100.00 ± 0.00	100.00 ± 0.00	100.00 ± 0.00	100.00 ± 0.00	100.00 ± 0.00
	II	B-E	99.00 ± 0.06	98.00 ± 0.08	98.50 ± 0.05	100.00 ± 0.00	100.00 ± 0.00	100.00 ± 0.00
	III	C-E	99.00 ± 0.06	99.00 ± 0.06	99.00 ± 0.04	99.00 ± 0.06	100.00 ± 0.00	99.50 ± 0.03
	IV	D-E	100.00 ± 0.00	100.00 ± 0.00	100.00 ± 0.00	100.00 ± 0.00	100.00 ± 0.00	100.00 ± 0.00
	V	A-D	100.00 ± 0.00	99.00 ± 0.06	99.50 ± 0.03	100.00 ± 0.00	100.00 ± 0.00	100.00 ± 0.00
	VI	AB-E	99.00 ± 0.06	99.00 ± 0.04	99.00 ± 0.03	100.00 ± 0.00	100.00 ± 0.00	100.00 ± 0.00
	VII	CD-E	97.00 ± 0.13	98.50 ± 0.05	98.00 ± 0.04	99.00 ± 0.06	100.00 ± 0.00	99.33 ± 0.03
	VIII	ACD-E	97.00 ± 0.13	99.00 ± 0.03	98.50 ± 0.05	100.00 ± 0.00	99.67 ± 0.02	99.75 ± 0.02
	IX	BCD-E	96.00 ± 0.13	99.67 ± 0.02	98.75 ± 0.05	99.00 ± 0.06	99.33 ± 0.03	99.50 ± 0.02
	X	ABCD-E	96.00 ± 0.13	99.75 ± 0.02	99.00 ± 0.03	99.00 ± 0.06	99.50 ± 0.02	99.60 ± 0.02
	XI	A-D-E	-	-	100.00 ± 0.00	-	-	100.00 ± 0.00
	XII	AB-CD-E	-	-	97.40 ± 0.07	-	-	99.00 ± 0.04
CHB-MIT	XIII	Non-seizure–Seizure	93.46 ± 0.12	92.83 ± 0.17	93.14 ± 0.07	95.70 ± 0.06	95.89 ± 0.10	95.79 ± 0.05

**Table 10:** The 20 most important features in the Bonn dataset.

Feature	Importance Score	Component	Domain	Description
Change_quantiles	0.0487	*IMF*_3_	Time	The absolute, average value of consecutive changes inside the corridor given by the quantiles 0.1 and 0.2
Agg_linear_trend	0.0430	*IMF*_2_	Time	A linear least-squares regression for values of the time series
Agg_linear_trend	0.0358	*IMF*_3_	Time	A linear least-squares regression for values of the time series
Svd_entropy	0.0352	*IMF*_1_	Entropy	Singular value decomposition entropy
FFT_aggregated_skew	0.0352	*IMF*_1_	Frequency	The spectral skew of the absolute Fourier transform spectrum
AR_coefficient	0.0336	*IMF*_1_	Frequency	The autoregressive coefficients
Percentage_of_reoccurring_datapoints_to_all	0.0272	Raw	Time	The percentage of unique values which appear more than once
Ratio_value_number_to_time_series_length	0.0230	Raw	Time	A factor which is 1 when all values in the time series appear only once, and below 1 otherwise
Agg_linear_trend	0.0204	Raw	Time	A linear least-squares regression for values of the time series
AR_coefficient	0.0165	Raw	Time	The autoregressive coefficients
AR_coefficient	0.0153	*IMF*_10_	Time	The autoregressive coefficients
Approximate_entropy	0.0143	*IMF*_1_	Entropy	Approximate entropy
FFT_coefficient	0.0141	*IMF*_4_	Frequency	The Fourier coefficients of discrete Fourier transform
Autocorrelation	0.0138	*IMF*_2_	Time	The autocorrelation of the specified lag (8)
Absolute_sum_of_changes	0.0137	*IMF*_2_	Time	The sum of the absolute value of consecutive changes
Change_quantiles	0.0113	*IMF*_2_	Time	The absolute, average value of consecutive changes inside the corridor given by the quantiles 0.8 and 0.0
Agg_autocorrelation	0.0108	Raw	Time	The value of an aggregation function over the autocorrelation
Autocorrelation	0.0102	Raw	Time	The autocorrelation of the specified lag (6)
Approximate_entropy	0.0098	Raw	Entropy	Approximate entropy
Number_CWT_peaks	0.0094	Raw	Time-frequency	The number of peaks that appear at enough width scales and with sufficiently high signal-to-noise ratio (SNR)

**Table 11:** The 20 most important features in the CHB-MIT dataset.

Feature	Importance Score	Component	Domain	Description
Change_quantiles	0.0527	*IMF_4_*	Time	The absolute, average value of consecutive changes inside the corridor given by the quantiles 0.2 and 0.4
Quantile	0.0423	Raw	Time	The 0.8 quantile of the raw signal
Agg_linear_trend	0.0419	*IMF*_4_	Time	A linear least-squares regression for values of the time series
Change_quantiles	0.0359	*IMF_5_*	Time	The absolute, average value of consecutive changes inside the corridor given by the quantiles 0.2 and 1.0
Change_quantiles	0.0206	Raw	Time	The absolute, average value of consecutive changes inside the corridor given by the quantiles 0.4 and 0.6
FFT_coefficient	0.0179	*IMF*_1_	Frequency	The Fourier coefficients of discrete Fourier transform
Quantile	0.0165	*IMF*_4_	Time	The 0.2 quantile of the raw signal
Number_peaks	0.0148	*IMF*_1_	Time	The number of peaks of at least support 1 in the signal
FFT_coefficient	0.0145	*IMF*_4_	Frequency	The Fourier coefficients of discrete Fourier transform
number_crossing	0.0083	*IMF*_1_	Time	The number of crossings of the signal on 1
Autocorrelation	0.0051	*IMF*_1_	Time	The autocorrelation of the specified lag (9)
Range_count	0.0046	*IMF*_2_	Time	Count observed values within the interval [−1, 1)
energy_ratio_by_chunks	0.0043	*IMF*_1_	Time	The sum of squares of chunks; 3 out of 10 chunks expressed as a ratio with the sum of squares over the whole time series
Permutation_entropy	0.0041	*IMF*_4_	Entropy	Permutation entropy
Change_quantiles	0.0040	*IMF*_2_	Time	The absolute, average value of consecutive changes inside the corridor given by the quantiles 0.0 and 0.6
FFT_coefficient	0.0039	*IMF*_3_	Frequency	The Fourier coefficients of discrete Fourier transform
FFT_coefficient	0.0036	*IMF*_7_	Frequency	The Fourier coefficients of discrete Fourier transform
Cwt_coefficients	0.0035	*IMF*_4_	Time-frequency	Continuous wavelet transform for the Ricker wavelet
Number_peaks	0.0035	*IMF*_3_	Time	The number of peaks of at least support 3 in the signal
Change_quantiles	0.0034	*IMF*_8_	Time	The absolute, average value of consecutive changes inside the corridor given by the quantiles 0.0 and 0.6
